# Supplementary material for: The choline-binding proteins PspA, PspC, and LytA of Streptococcus pneumoniae and their interaction with human endothelial and red blood cells
Source: Infect Immun. 2023 Aug 8;91(9):e00154-23. doi: 10.1128/iai.00154-23 (PMC10501214; doi:10.1128/iai.00154-23)
Supplement: Fig. S1 — Biofilm formation- Complementation of lytA mutant. [file iai.00154-23-s0001.pdf]

Supplementary material

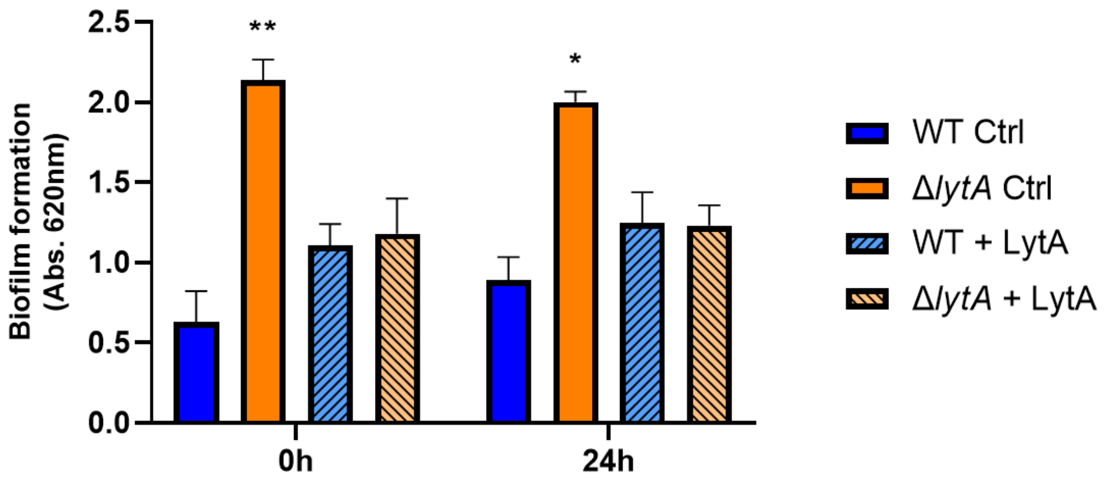

Figure S1- Biofilm formation- Complementation of *lytA* mutant. Biofilms were grown as described in Material & Methods.At two time points (either 0 or 24h), 5 µg/mL of recombinant LytA was added to the bacterial suspension / biofilms (patterned bars). The time indicated refers to the time of LytA addition and not to the age of the biofilms- Controls were kept without additional LytA (solid color bars). Biofilms were harvested at 48h growth and its quantification carried out via crystal violet staining. Significance is related to the corresponding WT. The experiment was repeated three independent times and the resulting mean and standard deviation are presented.
