# Supplementary material for: The choline-binding proteins PspA, PspC, and LytA of Streptococcus pneumoniae and their interaction with human endothelial and red blood cells
Source: Infect Immun. 2023 Aug 8;91(9):e00154-23. doi: 10.1128/iai.00154-23 (PMC10501214; doi:10.1128/iai.00154-23)
Supplement: Fig. S2 — Comparison of biofilm formation in different media. [file iai.00154-23-s0002.pdf]

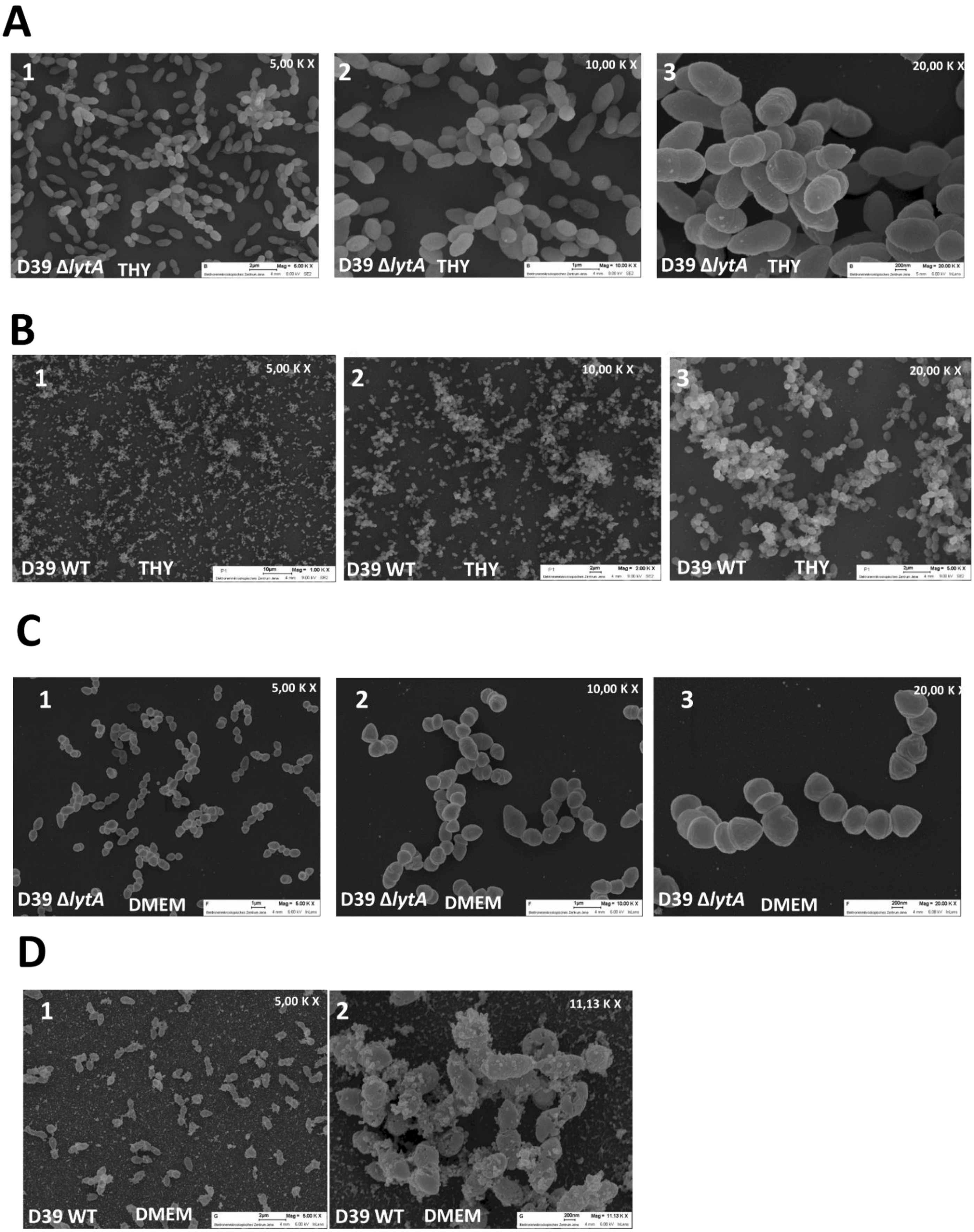

A
1
5,00 K X
D39 ΔlytA THY
2
10,00 K X
D39 ΔlytA THY
3
20,00 K X
D39 ΔlytA THY
B
1
5,00 K X
D39 WT THY
2
10,00 K X
D39 WT THY
3
20,00 K X
D39 WT THY
C
1
5,00 K X
D39 ΔlytA DMEM
2
10,00 K X
D39 ΔlytA DMEM
3
20,00 K X
D39 ΔlytA DMEM
D
1
5,00 K X
D39 WT DMEM
2
11,13 K X
D39 WT DMEM

Figure S2- Comparison of biofilm formation in different media. Representative SEM images of *lytA* mutant (**A**) or WT (**B**) biofilms formed on glass coverslips with THY media, with increasing magnifications (panels 1 to 3). (Representative SEM images of *lytA* mutant (C) or WT (D) biofilms formed on glass coverslips with DMEM, with different magnifications (panels 1 to 3 or 1 to 2, respectively).
