# Supplementary material for: The choline-binding proteins PspA, PspC, and LytA of Streptococcus pneumoniae and their interaction with human endothelial and red blood cells
Source: Infect Immun. 2023 Aug 8;91(9):e00154-23. doi: 10.1128/iai.00154-23 (PMC10501214; doi:10.1128/iai.00154-23)
Supplement: Fig. S3 — STRING Protein-Protein Interaction (PPI) Networks. [file iai.00154-23-s0003.pdf]

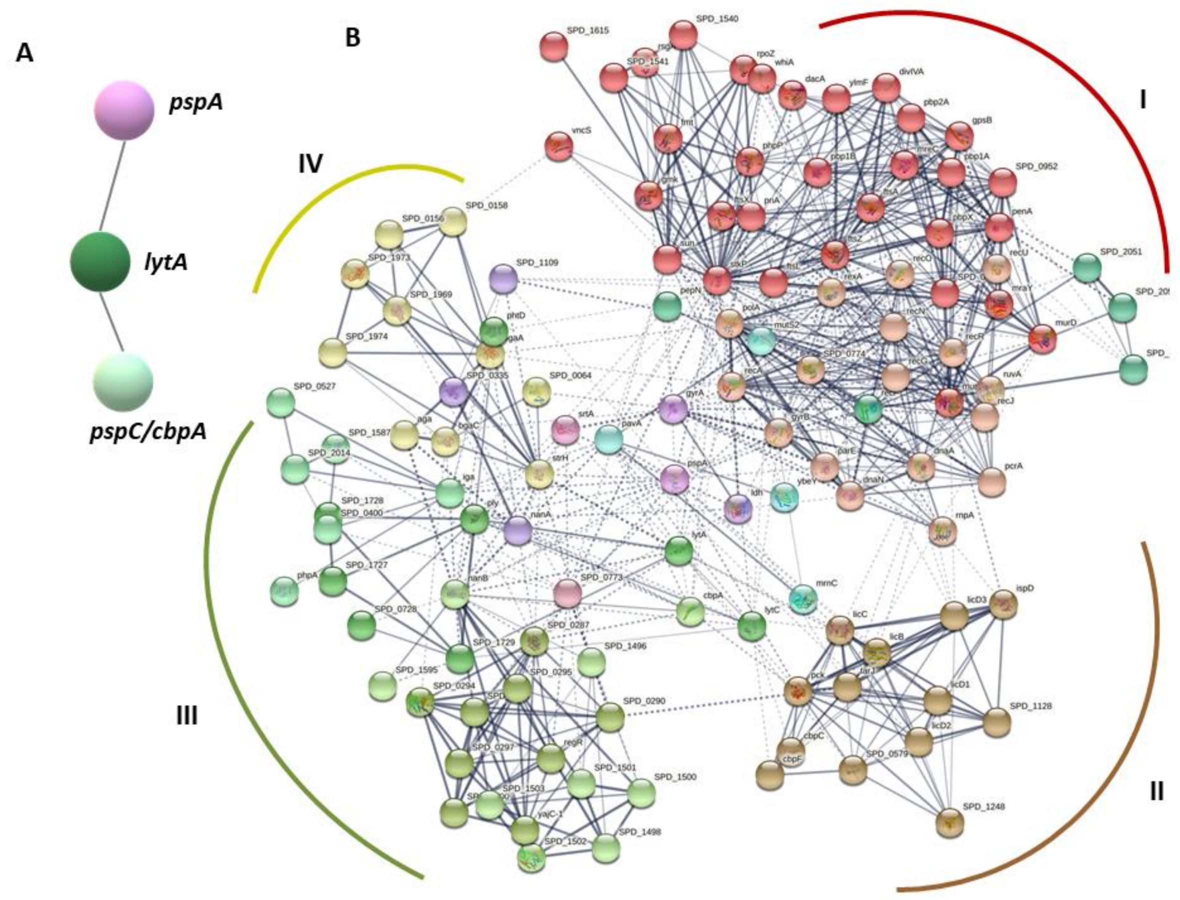

Figure S3- STRING Protein-Protein Interaction (PPI) Networks. (A)- Linear interaction network of the three studied CBPs, LytA, PspA and PspC (CbpA). Colorful circles or nodes represent the input proteins. (B)- PPI of interaction partners of PspA, PspC (CbpA) and LytA. Strength (confidence) of interaction is represented by thicker grey lines. Dashed lines represent interactions based in fewer criterions. The different colors are specific of different clusters (MCL clustering).
