# Supplementary material for: The choline-binding proteins PspA, PspC, and LytA of Streptococcus pneumoniae and their interaction with human endothelial and red blood cells
Source: Infect Immun. 2023 Aug 8;91(9):e00154-23. doi: 10.1128/iai.00154-23 (PMC10501214; doi:10.1128/iai.00154-23)
Supplement: Table S1 — Influence of each strain on pneumococcal pathogenic features. [file iai.00154-23-s0005.pdf]

| Feature | D39 | Δ*pspA* | Δ*pspC* | Δ*lytA* |
|---|---|---|---|---|
| Biofilms | ↓ | ↓ | ↓ | ↑ |
| Epithelial metabolism | ↓ | ↑ | ↑ | ↑ |
| Haemolysis | ↑ | ↓ | ↓ | ↓ |
| Endothelial PECAM-1 expression | ↑ | ↓ | ↓ | ↓ |

**Table S1- Summarizing table of the influence of each strain on pneumococcal pathogenic features.** Upwards arrow indicates an increase activity, downwards arrow indicate a decrease activity.
